# Personal genomics: Where are we now?

**DOI:** 10.1016/j.atg.2016.01.010

**Published:** 2016-02-01

**Authors:** Misha Angrist

**Affiliations:** Duke University Initiative for Science & Society, Social Science Research Institute, and Sanford School of Public Policy, Durham, NC, USA

*Where are we now?*

*The moment you know*…

David Bowie (1947–2016)

When editor-in-chief Carol Isaacson Barash asked me to edit a special issue of *Applied* & *Translational Genomics* dedicated to personal genomics, I was excited but not sure where to begin. I had tried to chronicle the early, heady days of individuals gaining access to their own genomic information circa 2007–2010 (Angrist, 2010), but somehow so much and so little have happened since then: was the narrative to be one of seismic shift (thanks to increasingly cheap genotyping/sequencing and the concomitant growth of personal genomics databases) or boring stasis/retrenchment (thanks to still-missing heritability and regulatory clampdowns)?

Consequently, I opted to refrain from imposing some kind of overarching theme and rather, approached it as a “hypothesis-free” experiment. We solicited papers from a range of researchers, scholars and patient activists (and the broader genomics community) with the only prerequisite being that they submit something about which they were passionate and could speak to with some authority, whether that authority stemmed from empirical data or personal experience (or both).

In hindsight it seems clear that, with respect to personal genomics, the answer to the late great Mr. Bowie's question is manifold: like a promiscuous electron, we are in several places at once, some promising and some problematic (and some both).

## 1 The perceived value of personal genomics

The prospect of having access to one's own DNA information brought with it sometimes heated debate about what practical purposes, if any, such information was good for. Most of us agreed—and the data showed—that it was not dangerous (Bloss et al., 2013). Okay, but could it, by itself, be predictive of health outcomes for complex diseases? Many of us < cough > wanted to believe that it could be. But nearly a decade and well over a million people into this, we are left to conclude that, with few exceptions, the predictive power of chip-based personal genomics for complex traits amounts to fairly weak sauce (Jordan, 2014, Wray et al., 2013).

But if that is the case, then why do users continue to purchase direct-to-consumer genetic tests (Servick, 2015)? (And why have the largest commercial players in DNA sequencing technology and genealogy made major strategic moves toward DTC genomics (Anonymous, 2015, Hernandez, 2015)?) In their paper Mauro Turrini and Barbara Prainsack address the consumer paradox head on (Turrini and Prainsack, 2016). They broach potential health implications and the fraught notion of “personal utility” (e.g., the purchase of longterm care insurance upon discovering one's greater risk for Alzheimer's disease (Taylor et al., 2010)). Turrini and Prainsack eschew discussion of the tired sawhorse of “actionability” in favor of an exploration of the intrinsically social nature of personal genomic data and its relationships to family, ancestry and the research community.

Elsewhere, Laura Mählmann, Effy Vayena and colleagues describe the results of a survey of Swiss adults over the age of sixty (Mählmann et al., 2016). Their findings reveal a lingering generational divide: only one-third of respondents were familiar with personal genomics, while nearly half were not interested in undergoing such testing because they thought the results might be worrisome or they harbored concerns about the validity of the tests. Of those who wanted to participate in personal genomic testing, the number one reason for wanting to take part was to learn about their own health risks, followed by a desire to contribute to research. Turrini and Prainsack flagged the latter as well (and 23andMe has certainly noticed), yet it is a motive that often goes overlooked and is deserving of more attention.

## 2 The uncertain bargain

Internet commerce has been crucial to the rise of commercial DTC genomics. The ability to click—and pay—seamlessly means that it is much easier to build a database of more than a million customers than it would be if one had to rely on the postal service or brick-and-mortar stores.

But what exactly have we million agreed to by clicking? Beyond sending our credit card information in exchange for parsed genotypes, what does accepting a DTC company's terms of service actually mean? Two papers in this issue reveal why the answer is not always clear.

Andelka Phillips offers an overview of the DTC genetic testing landscape (which now extends far beyond health-related and ancestry testing to forensics, diet, infidelity and matchmaking) and the regulatory challenges posed by it (Phillips, 2016). She describes the industry's reliance on so-called wrap contracts that, in some cases, do not even require the consumer to open the link to the actual contract, let alone read it, before clicking “I agree.” Of course most of us decline to read terms of service anyway (Böhme and Köpsell, 2010). But in an increasingly hackable post-Snowden world, do we do so at our peril?

The work of Emilia Niemiec and Heidi Howard suggests that even a close reading of such terms might not always lead to informed purchasing decisions. The authors scrutinized the websites of four North American companies offering whole-genome and/or whole-exome sequencing to consumers (Niemiec and Howard, 2016). They found that while these companies have upped their game with respect to data security measures, they remain less than fully transparent about their policies on data and sample storage, secondary use and sharing/disclosure. Caveat emptor.

## 3 Who gets to know: access and disclosure

While large-scale sequencing and genotyping have become routine, unfettered sharing of the information with those individuals from whom the samples and data came has not. As I′ve written elsewhere, this is unfortunate (Angrist, 2011, Angrist, 2016). Not only does it perpetuate notions of genetic exceptionalism, it belies the ethos of partnership that, in the dawning era of precision medicine, is suddenly au courant. This frustration is palpable in the commentary an anonymous author sent me, in which she describes the gauntlet she had to run for a period of months before getting access to her extended exome report (Anonymous, 2016).

Which is why places like Geisinger Health System deserve credit for walking the return-of-results walk. In their commentary, Andy Faucett and Dan Davis recount the origins of Geisinger's decision to share secondary findings with patients undergoing whole-genome/whole-exome sequencing (Faucett and Davis, 2016). Unlike other large-scale genomic initiatives (I′m looking at you, publicly funded Million Veteran Program (Gaziano et al., 2015, Kaufman et al., 2012)), when patients and participants said they wanted their genomic results, Geisinger actually listened. And perhaps the tide is turning: As of early 2016, 23andMe was set to launch a program whereby academic researchers could return the company's product to their research participants (J. Hagenkord, personal communication).

Of course, the implications of genetic information are not limited either to isolated individuals or to health per se. Particularly if it refutes presumptive biological relationships, DNA can upend family dynamics in profound and sometimes tragic ways (Doe, 2014). This has been the primary hitherto justification for genetics professionals not to disclose misattributed paternity to patients—indeed nondisclosure has been the norm for many decades. But in their bold and provocative paper, Laura Hercher and Leila Jamal make compelling arguments for rethinking the status quo (Hercher and Jamal, 2016). Not only is genetic testing more sophisticated, so too are those undergoing such testing. As the authors note, “In an age where we are counseling our patients to empower themselves by understanding their own health risks and susceptibilities, we cannot rely on misdirection and sleight of hand to hide pertinent facts fundamental to their biological selves.” (Hercher and Jamal, 2016).

## 4 Self-starters: exploration, alienation and determination

For patient activists, the stakes—often a desperately ill child with an unknown causative genetic variant and/or limited treatment options—are obviously much higher. For them, personal genomics is excruciatingly personal.

In separate commentaries, both Terry (2016) andCollins (2016) point up the challenges faced by parents of children with serious rare conditions. Initially, at least, neither author wished to go around the system but rather to work within it—to champion it and help it to grow…yet time and again both have found themselves ignored or even thwarted by it. Terry describes her shock and horror at discovering a human genetics ecosystem that often placed professional advancement ahead of patient needs (Terry, 2016). For her part, Collins laments that the same enthusiasm and resources dedicated to sequencing are not brought to bear on what comes *after* the diagnosis (Collins, 2016). Both she and Terry are determined to “raise the stature of phenotype,” in part by aggregating parent observations of their children (another word for this is “data”). Will the medical community finally learn to heed them?

Hugh Rienhoff's story is well known to many, having been told in the pages of *Nature* (Maher, 2007) and *Wired* (Koerner, 2009), among other places. In this issue he offers a first-person account of an arduous decade-long diagnostic odyssey and of the many heroic people he enlisted and the sometimes maddening obstacles he encountered en route to identifying his daughter Bea's *TGF*β*3* mutation (Rienhoff, 2016). Rienhoff's unique background as a former medical geneticist and current head of a startup developing therapies for genetic diseases affords him a singular perspective on the art and science of diagnostic sleuthing and the biology underlying Mendelian conditions. And he clearly recognizes the varied incentives that drove people to help his family and, occasionally, to turn away from them.

Rienhoff also reminds us of a couple of other things. The first is how hard it is to navigate the terrain of the undiagnosed under any circumstances: having a personal stake and access to intellectual and technological resources can shorten the time to answers, but the road remains long and circuitous. As a colleague of mine likes to say, these studies are never over.

The second is about how we view ourselves. Many of us have championed access to genetic information for a long time for many valid reasons: for our health, yes, but also for reproductive decisionmaking, STEM education and, as mentioned, as a means to participate in and contribute to research. But increasingly we recognize its limitations. However vivid our deterministic dystopian fantasies, without phenotype, environment and trait data, a genome is a pathetic proxy for what makes a human being.

So as we celebrate quantum advances in sequencing, wring our hands over missing heritability and inadequate databases, negotiate fraught questions of genetic access and disclosure, and embark on increasingly ambitious “moonshots,” perhaps we might also the most adaptive phenotype of the last decade: humility.

As Hugh Rienhoff says of his daughter, “She is so much more than her DNA.”

## References

[bb0005] Angrist M. (2010).

[bb0010] Angrist M. (2011). You never call, you never write: why return of 'omic' results to research participants is both a good idea and a moral imperative. Pers. Med..

[bb0015] Angrist, M. Patients Should Receive Study Results. *Genome Magazine* (11 January 2016).

[bb0020] Anonymous (2015). Illumina opens Helix genomics store. Nat. Biotechnol..

[bb0025] Anonymous (2016). Exomes for we but not for thee. Applied & Translational Genomics.

[bb0030] Bloss C.S., Wineinger N.E., Darst B.F., Schork N.J., Topol E.J. (2013). Impact of direct-to-consumer genomic testing at long term follow-up. J. Med. Genet..

[bb0035] Böhme R., Köpsell S. (2010).

[bb0040] Collins C. (2016). Phenotype with a side of genotype, please: patients, parents and priorities in rare genetic disease. Applied & Translational Genomics.

[bb0045] Doe G. (2014). Vox.

[bb0050] Faucett W.A., Davis F.D. (2016). How Geisinger made the case for an institutional duty to return genomic results to biobank participants. Applied & Translational Genomics.

[bb0055] Gaziano J.M. (2015). Million Veteran Program: a mega-biobank to study genetic influences on health and disease. J. Clin. Epidemiol.

[bb0060] Hercher L., Jamal L. (2016). An old problem in a new age: revisiting the clinical dilemma of misattributed paternity. Applied & Translational Genomics.

[bb0065] Hernandez D. (2015). Fusion.

[bb0070] Jordan B. (2014). Genes and non-mendelian diseases: dealing with complexity. Perspect. Biol. Med..

[bb0075] Kaufman D., Bollinger J., Dvoskin R., Scott J. (2012). Preferences for opt-in and opt-out enrollment and consent models in biobank research: a national survey of Veterans Administration patients. Genet. Med..

[bb0080] Koerner B. (2009). DIY DNA: one father's attempt to hack his daughter's genetic code. Wired.

[bb0085] Maher B. (2007). Personal genomics: his daughter's DNA. Nature.

[bb0090] Mählmann L., Röcke C., Brand A., Hafen E., Vayena E. (2016). Attitudes towards personal genomics among older Swiss adults: an exploratory study. Applied & Translational Genomics.

[bb0095] Niemiec E., Howard H.C. (2016). Ethical issues in consumer genome sequencing: use of consumers' samples and data. Applied & Translational Genomics.

[bb0100] Phillips A.M. (2016). Only a click away – DTC genetics for ancestry, health, love…and more: a view of the business and regulatory landscape. Applied & Translational Genomics.

[bb0105] Rienhoff H.Y.J. (2016). Reflections on my daughter's DNA. Applied & Translational Genomics.

[bb0110] Servick K. (2015). Can 23andMe have it all?. Science.

[bb0115] Taylor D.H. (2010). Genetic testing for Alzheimer's and long-term care insurance. Health Aff..

[bb0120] Terry S.F. (2016). Life as a numerator: putting the person in personal genomics. Applied & Translational Genomics.

[bb0125] Turrini M., Prainsack B. (2016). Beyond clinical utility: the multiple values of DTC genetics. Applied & Translational Genomics.

[bb0130] Wray N.R. (2013). Pitfalls of predicting complex traits from SNPs. Nat. Rev. Genet..

